# Supplementary figures and images for: Human-like dissociations between confidence and accuracy in convolutional neural networks
Source: PLoS Comput Biol. 2024 Nov 14;20(11):e1012578. doi: 10.1371/journal.pcbi.1012578 (PMC11594416; doi:10.1371/journal.pcbi.1012578)

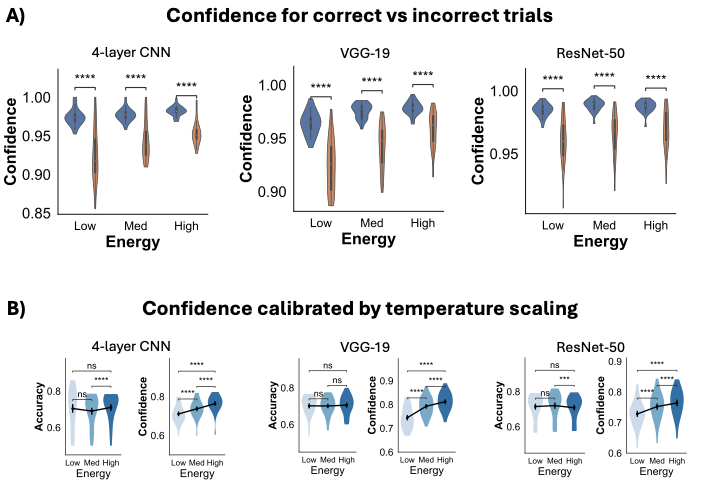

Supplement: S1 Fig — (A) We tested whether the CNN’s confidence could reliably discriminate between correct and incorrect choices for the different energy conditions. We found that for all stimulus energy levels and all three networks (4-layer CNN, VGG-19 and ResNet-50) confidence was higher for correct choices compared to incorrect choices, confirming that the networks’ confidence reliably tracked their accuracies. (B) CNNs tend to be overconfident and generate confidence values greater than their accuracies. Therefore, we tried to calibrate the CNNs’ confidence using a post-processing method called “temperature-scaling” [29] which uses a parameter (T) that can scale the network’s confidence to match its accuracy. The network’s output in logits (z) is scaled before the sigmoid transformation such that zscaled=zT. Confidence is then computed as 11+e−zT and therefore, when T>1, the network’s confidence gets scaled down. We found that temperature scaling indeed reduced overconfidence in all networks (4-layer CNN: confidence decreased from 0.968 to 0.737; VGG-10: confidence decreased from 0.964 to 0.783, ResNet-50: confidence decreased from 0.982 to 0.748). However, for all networks we still robustly observed the confidence-accuracy dissociations. (TIFF) [file pcbi.1012578.s001.tiff]

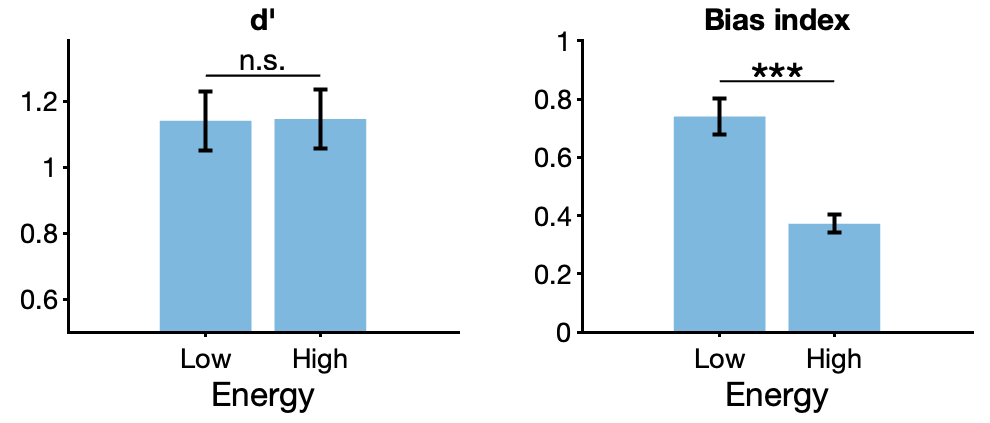

Supplement: S2 Fig — Herce Castañón et al. (2019) [13] showed that when subjects are asked to judge the mean orientation of an array of Gabor patches, they exhibit a range of suboptimal behaviors. Specifically, Herce Castañón et al. [13] tested how energy manipulation interact with changes in stimulus base rates by measuring the shifts in subjects’ decision criteria in response to changes in stimulus frequencies for each energy condition. The location of the decision criteria was computed from the SDT-based measure of response bias (c). The shift in criteria in response to changes in stimulus base rates was quantified as cS1−cS2 where cSi refers to the criterion in the condition where stimulus i is more frequent. Herce Castañón et al. [13] found that subjects showed significantly smaller shifts in their criterion in response to base rate changes in the high-energy condition, compared to the low-energy condition. They explained these effects by proposing that observers are blind to the noise arising from their own cognitive computations, thus resulting in failure to account for the higher levels of uncertainty arising from high-energy stimuli. Here, we show via simulations that the signal-and-variance-increase hypothesis can also explain their observed effects. We simulated the effect of stimulus energy manipulations in an SDT model where higher stimulus energy led to increase in signal (μ) as well as variance (σ) of the stimulus evidence distributions. We generated 50 simulations by sampling individual SDT parameters: the signal, μ, and the decision criterion, c, from Gaussian distributions. Specifically, in the low-energy condition μlow∼N(1,.5) and in the high-energy condition μhigh = N(2,1), such that the two distributions would lead to equal sensitivity but the high-energy condition features higher variance of the evidence distributions. Similarly, the decision criterion for individual simulations was sampled from c∼N(0,.25). For the base-rate manipulations, we assumed that an increa [file pcbi.1012578.s002.tif]
