# Supplementary material for: Human-like dissociations between confidence and accuracy in convolutional neural networks
Source: PLoS Comput Biol. 2024 Nov 14;20(11):e1012578. doi: 10.1371/journal.pcbi.1012578 (PMC11594416; doi:10.1371/journal.pcbi.1012578)
Supplement: Attachment — Submitted filename: Rebuttal.docx [file pcbi.1012578.s005.docx]

Dear Dr. Doumas,

Thank you for giving us the opportunity to revise and improve our manuscript. We also want to thank all the reviewers for their helpful comments and suggestions. We have now edited the paper extensively to address concerns from Reviewers 2 and 3 regarding the generalizability of our conclusions. Specifically, we have removed all general claims that CNNs allow us to distinguish between low- and high-level processes and restricted our conclusions to the specific hypotheses we test. We have also clarified that our findings only test the necessity of cognitive mechanisms such as the positive evidence bias and noise-blindness and cannot be used as evidence against these mechanisms. Secondly, as per Reviewer 1’s suggestion, we have extended our analyses of meta-d’-d’ dissociations to the VGG-19 and ResNet-50 models. Finally, following Reviewer 3’s suggestion, we have included additional analyses to test the calibration and resolution of confidence in our models. We believe our paper is much improved as a result of these additions. We provide a point-by-point response to the reviewers’ comments below.

Sincerely,

Medha Shekhar and Dobromir Rahnev

**Reviewer #1**

***The current work presents a series of simulations testing the hypothesis that dissociations between confidence and accuracy found in the psychological literature can be explained by low level changes in the separation and variance of perceptual representations. The overall strategy consisted of training deep neural networks (DNNs) to classify Gabor patches stimuli into clockwise and counterclockwise categories and testing on stimuli groups varying on energy (contrast plus variability) in three main experiments. The results showed that three different models (a custom 4-layer CNN, a VGG-19, and a ResNet-50), showed a dissociation between accuracy and confidence, with accuracy being constant while confidence increased with energy. I think this is an important work in perceptual decision-making as it helps to disentangle a current debate through simulations with image computable DNNs. I especially liked the rigorous experimental approach taken by the authors (see comments below). However, I do have some concerns that need to be addressed before I can recommend this manuscript for publication, which I detail in the following:***

We thank the reviewer for the positive and helpful comments.

***Section “Different stimulus features selectively influence the separability and spread of internal evidence distributions”:
• How were the main 3 experiments conducted? Before this section it seemed that Energy was a single independent variable in the experimental design of the 3 main studies, but in this section, it is revealed that, in fact, Contrast and Variability were also factors. Were these groups added to the groups of experiments 1-3 for comparison or experiments 1-3 had always Contrast and Variability as independent variables that were combined to generate the high energy condition? From the writing this is unclear.***

We apologize for the confusion. We want to clarify that the “energy” of a stimulus always consists of two independent variables being manipulated simultaneously in the same direction. For example, the energy of a stimulus can be increased by jointly increasing both its contrast and noise levels. Energy cannot be manipulated by changing any single variable. However, in the later section, we test how the networks respond to changes in each variable separately, while keeping the other variable fixed. We have now clarified this point in the main text (in the Introduction and Results) and paste the relevant section below:

Introduction

… A particular type of confidence-accuracy dissociation has been induced by stimulus manipulations referred to as “energy manipulations” (Gao et al., 2023; Zylberberg et al., 2012). Energy manipulations consist of two independent stimulus features being simultaneously altered in the same direction. Specifically, stimulus energy can be increased by simultaneously increasing stimulus features that provide evidence for the correct choice (e.g., by enhancing stimulus contrast or motion coherence) and increasing stimulus features that provide evidence for the incorrect choice or simply add to the uncertainty of the stimulus (e.g., by enhancing strength of evidence for the distractor stimulus or the variance in the signal).

Results

…. In each experiment, we manipulated the energy of the stimulus by simultaneously varying two independent stimulus features in the same direction: the contrast of the stimulus and its variability (in Experiments 1 and 3) or the contrast of the stimuli associated with the correct and incorrect choices (in Experiment 2).

***Section “CNNs can reproduce dissociations between type-1 and type-2 sensitivity typically regarded as evidence for the positive evidence mechanism”:
• Why was this study only run with the custom 4-layer network? Since the results are based on the activation of the output (single unit with sigmoid activation), it should be possible to run the study with the VGG-19 and ResNet-50 models, especially considering that the main result, the cross-over between the S1 and S2 responses in their meta-d’ and d’ relationship, was not found for Experiment 2. I strongly encourage the authors to run with the VGG-19 and ResNet-50 models to check the robustness of their results in this section.***

We thank the reviewer for this suggestion. We have now performed these analyses for the VGG-19 and ResNet-50 models for Experiments 1-3. Overall, we find that all the networks are able to reproduce this dissociation in at least 2 out of 3 experiments. While the 4-layer CNN produces this dissociation for Experiments 1 and 3, VGG-19 and ResNet-50 produce this dissociation for Experiments 1 and 2, suggesting that is indeed possible to generate these dissociations in the absence of the PE mechanism. However, it is likely that the emergence of this effect depends on specifics of the stimuli and the network. We now paste the results from these analyses below:

To further test the necessity of the positive evidence mechanism in explaining confidence, we assessed whether CNNs lacking this mechanism can also reproduce the dissociation observed in Maniscalco et al. (2016). We simulated the above task paradigm for our previously trained networks across the three experiments (as done by Webb et al., 2023) and found that all the networks were indeed able to reproduce a clear dissociation between meta-d’ and d’ for at least two out of the three experiments (Figure 5). While the 4-layer CNN produced this dissociation for Experiments 1 and 3, VGG-19 and ResNet-50 produced these dissociations for Experiments 1 and 2. Specifically, meta-d’ increases with d’ for trials with 〖"S〗_2 " responses (where the contrast of S_2 varies across trials), but meta-d’ decreases with d’ for trials with 〖"S〗_1 " responses (the contrast of 〖"S〗_1 remaining fixed across trials), producing the distinct cross-over signature shown by Maniscalco et al. (2016). It is possible that unlike the confidence-accuracy dissociations examined above, the meta-d’/d’ dissociations are more sensitive to the specific interactions between the stimuli and the networks. Nevertheless, our findings demonstrate that for at least for some stimulus manipulations and networks, the positive evidence mechanism is unnecessary to explain effects that have typically been considered as evidence for this mechanism.

**Figure 5. Dissociations between meta-d’ and d’ in 4-layer CNNs.** We tested the three networks (4-layer CNN, VGG-19 and ResNet-50) on the task paradigm from (Maniscalco et al., 2016) which demonstrated a dissociation between d’ and meta-d’ under certain conditions. The contrast of one stimulus category ${(S}_{1})$remains fixed while the contrast of the other stimulus is increased in discrete steps ${(S}_{2}).$Meta-d’ increases with d’ as expected for trials in which the observer responds ${"S}_{2}",$but meta-d’ decreases with d’ while for trials where the observer responds ${"S}_{1}"$. This behavioral effect is predicted by a model incorporating the positive-evidence bias. Here, we simulated this task paradigm for Experiments 1-3. The responses generated by our networks show that they can indeed generate the meta-d’/d’ dissociations observed in humans for at least 2 out of 3 experiments. While VGG fails to to reproduce this behavior for Experiment 2, VGG-19 and ResNet-50 fail to produce this behavior for Experiment 3, suggesting that these dissociations may depend on specific interactions between the stimuli and the networks.

***Section “CNNs as models for understanding human vision”:
• While it is true, that many have argued that DNNs can provide insights into the workings of the visual system, many of the papers cited by the authors in this regard fail to emphasize what I think is one of the strengths of the current work: the manipulation of independent variables in order to contrast theoretically motivated hypothesis. Bowers et al. (2023a) have empathized the prevalent lack of experimental manipulations on deep learning research. Furthermore, Bowers et al. (2023b) have argued for the need of severe testing of DNNs as models of human cognition. I think the discussion of the current work would benefit from making its methodological strengths in comparison to correlational DNNs studies more explicit.***

We thank the reviewer for this helpful suggestion. We agree that one of the main strengths of this study is the manipulation of independent variables that show us how CNNs represent individual stimulus features and how these representations influence their behavior. In turn, these findings allow us to test CNNs against theories of human perception and can inform the debate on the usefulness of CNNs as models of human vision. We now include a paragraph in the Discussion where we emphasize this aspect of our study and paste the paragraph below for reference:

… Our study can also help inform the debate on whether CNNs can serve as useful models of human vision. Bowers et al. (2023) argue against the notion that CNNs can be applied as models of human perception since these models have not been adequately tested on their ability to account for findings in cognitive science research. Although CNNs perform visual classification tasks with high levels of accuracy, it is not yet understood whether these networks process stimulus features in ways similar to humans. Bowers et al. argue that to establish such similarity in processing, it is important to test CNN’s behavior on manipulations of independent stimulus features and compare their behavior to humans on psychological tasks. In this study, we follow a similar logic as we manipulate stimulus features such as contrast and variance and find that CNNs indeed respond similarly to humans under the specific stimuli tested here. Further, these manipulations change the properties of the CNNs’ evidence distributions in a way that is consistent with the predictions of an empirically validated model of perception, suggesting that, in the case of the stimuli tested here, a common mechanism can possibly explain the behavior of both humans and ANNs.

**Reviewer #2**

***The authors investigate whether CNN architectures can help to distinguish between two different possible mechanisms that might explain why stimulus energy (e.g., contrast, variability) impacts confidence ratings (but not accuracy) in a visual discrimination task. One hypothetical mechanism is a high-level ‘positive evidence heuristic’ account and another is framed as the result of low-level visual processing. Because CNNs do not have a mechanism for confirmatory bias, the authors think that these networks can be useful for deciding between the two possibilities.

The work is likely of interest to people using signal detection modeling to try to understand visual discrimination behavior. The main contribution of the paper is an existence proof using CNNs that a confirmatory bias heuristic is not required to explain the dissociation between confidence and accuracy in visual processing. This seems to be related to the Webb et al. (2023) paper that the authors cite.

However, the paper is framed as a project that directly investigates the contributions of high and low level processing to this dissociation effect in people, using CNNs as models of the human visual system. Here the paper is not successful in its aims. There are several problems with this framing of the work that mostly stem from the choice of CNNs as a model. While the existence proof that the prior SDT modeling research was flawed in its conclusions is a nice contribution, that does not mean that all types of high level processing are eliminated as explanations for, or contributions to, the effect in question in the human visual system.

The authors show in the paper that manipulating stimulus energy at the input to a CNN causes changes in the distribution of patterns on the output layer in ways that are consistent with the dissociation effect between confidence and accuracy. However, even if we were to take CNNs at face value as models of human vision (which we definitely should not for reasons elaborated below), this work is not able to speak to the level of processing in any general sense. CNNs, including the ones used in this research, are ambiguous in terms of where representations lie in the visuo-cognitive processing stream. The input to a CNN is probably something like LGN. The output of a typical CNN is often an object label, which should correspond to late vision at the earliest, or according to some accounts, language or other cognitive processes that probably involve prefrontal cortex. So demonstrating this effect using a CNN does not isolate the mechanism to “low level” processing in any way. This claim is an overinterpretation of their results.***

We thank the reviewer for the careful and thorough feedback. We agree with the reviewer’s point that the claims regarding CNNs allowing us isolate low-level processes from cognitive or high-level processes is too broad and given that CNNs cannot yet be considered as good model the human vision, our results do not allow us to draw conclusions about the processes occurring in the brain. Following the reviewer’s suggestion, we have now removed all claims about CNN’s allowing us to distinguish between low- and high-level processes. We now limit our conclusions to the specific mechanisms (the positive evidence bias, noise blindness and signal-and-variance-increase) we test in this study. Additionally, we also emphasize the point that our results cannot falsify the positive evidence bias and noise-blindness mechanisms in humans. Rather, we only show that these mechanisms, in spite of their popularity, are not necessary to explain these observed dissociations. We have now made edits throughout the paper to reflect these changes. Below, we paste some of the edited portions from the Abstract and Introduction as examples:

Abstract

These findings cast doubt on the necessity of the positive evidence heuristic to explain human confidence and establish CNNs as promising models for testing cognitive theories of human behavior.

Results

… Here, we use convolutional neural networks (CNNs) to distinguish between these competing explanations of the confidence-accuracy dissociations induced by stimulus energy manipulations. Standard CNNs give confidence using the same signal as the decision, and thus do not use the positive evidence heuristic (where decision-incongruent evidence is ignored during confidence computations). They also give confidence without building internal models of the task. Therefore, if the positive evidence heuristic (Koizumi et al., 2015; Maniscalco et al., 2016; Peters et al., 2017; Samaha et al., 2016) or inference from suboptimal internal models (Herce Castañón et al., 2019; Zylberberg et al., 2014) are indeed necessary for these dissociations, these networks should fail to mimic human behavior. On the other hand, if confidence-accuracy dissociations arise from a signal-and-variance increase based on inherent stimulus or task characteristics (Fetsch et al., 2014; Morales et al., 2015; Rahnev et al., 2011, 2013; Rahnev, Maniscalco, et al., 2012; Zylberberg et al., 2016), we can expect neural network models to reproduce human behavior. An additional advantage of CNNs is that, unlike humans, we can directly probe the network’s internal representations and understand the mechanisms underlying their behavior.

…These results demonstrate that CNNs exhibit human-like dissociations between confidence and accuracy, showing that it is indeed possible for networks to generate these behaviors in the absence of mechanisms such as the positive evidence heuristic and cognitive inference. Importantly, these observations shed light on the possibility of common mechanisms driven by external features of the environment underlying the behavior of biological and artificial neural networks.

***Another issue with the CNN architecture is that CNNs are strictly feed forward, and the human visual system is not, with large numbers of back projections from prefrontal cortex and lateral connections at all levels of representation. There is broad evidence in the literature of continuous interactions of top-down, bottom-up, and lateral effects. By virtue of their architecture, CNNs cannot capture the fact that almost nothing in human vision is strictly bottom-up or top-down. What is cognitively penetrable at any level of representation also varies. The dichotomy between low-level and high-level processing that the paper (and perhaps the SDT field?) sets up seems to be behind what is known in the visual cognition literature.***

We agree that we cannot claim to have isolated the bottom-up visual processes in humans using CNNs because bottom-up processing in humans is influenced by top-down and lateral effects. We have therefore removed the claims regarding CNNs allowing us to isolate bottom-up effects. As mentioned in the previous response, we have limited our conclusions to the specific mechanisms tested in the study such as the positive evidence bias and noise-blindness.

***There are also some issues with the way a CNN and its representations are described in the paper, with some confusion about what a network’s ‘representations’ are (as opposed to mathematical or statistical characterizations of input or output data). For example, the authors refer to patterns of activity that they have characterized on the output layer of the CNN using a signal detection framework as ‘internal representations’. I think this description is misleading because CNNs do have actual internal representations (even if they are incomprehensible).***

Following the reviewer’s suggestion, we have now removed the term “internal representations” from the paper and replaced it with the term “distribution of activations” or “distribution of evidence” to refer to the distribution of networks’ activations in the final layer in a way that is independent on SDT terminology.

***If the paper could be reframed to speak to the issues in SDT modeling, and resist over claims about CNNs being good models of human visual cognition, it would be stronger. Late in the paper, in the discussion, the core issues about modeling the phenomenon are more clear.***

We thank the reviewer for the suggestion. We have now removed claims implying that CNNs allow us to model human vision. We have focussed our conclusions on the main issue that current explanation of energy-induced confidence-accuracy dissociations assume the necessity of confidence heuristics or cognitive inference without fully testing all viable alternatives. We have also emphasized the point that we are not claiming that CNN are good models of human vision. We only use them to test whether certain assumptions contained in cognitive models of confidence are necessary to generate observed behavior.

***Some of the statistics reporting needs to be cleaned up. A few sections could use editing for clarity or readability.***

We have edited our statistics reporting following the reviewer’s suggestions. We include a point-by-point response to their comments below.

***Please see specific notes below by page:
Page 6 (abstract)
The abstract could use some more context. Going in cold, I did not know from the abstract or title that this work was related to SDT modeling.***

We thank the reviewer for the suggestion. We have now edited the abstract and the Introduction to specify that the mechanisms we test are derived from cognitive modelling using the SDT framework. We paste the relevant section below:

Abstract:

… The most common explanation for this effect, derived from cognitive modeling, is the positive evidence heuristic where confidence neglects evidence that disconfirms the choice.

Introduction:

… Explanations of these types of dissociations typically invoke cognitive mechanisms or heuristics that are instantiated and tested using modelling frameworks such as signal detection theory (SDT; Green, D.M. and Swets, 1966).

… On the other hand, a simpler, SDT-based explanation for these effects has also been posited. According to this explanation, energy manipulations lead to changes in the distributions of evidence at the perceptual level, leading to higher confidence (Fetsch et al., 2014; Morales et al., 2015; Rahnev et al., 2011, 2013; Rahnev, Maniscalco, et al., 2012; Webb et al., 2023; Zylberberg et al., 2016).

***Page 7

Paragraph 2:
Remove the ‘s’ from dissociations.
Not sure what "strength of discomfirming evidence" means in this context, and there are no examples/citations.
In general, paragraph 2 could use some finessing for clarity and readability.***

We have modified this paragraph to more directly state that “the strength of disconfirming” refers to the strength of evidence in favor of the distractor stimulus. We have also clarified the definition of energy manipulations and edited the section to improve clarity. We paste the section below for reference:

A particular type of confidence-accuracy dissociation has been induced by stimulus manipulations referred to as “energy manipulations” (Gao et al., 2023; Zylberberg et al., 2012). Energy manipulations consist of two independent stimulus features being simultaneously altered in the same direction. Specifically, stimulus energy can be increased by simultaneously increasing stimulus features that provide evidence for the correct choice (e.g., by enhancing stimulus contrast or motion coherence) and increasing stimulus features that provide evidence for the incorrect choice or simply add to the uncertainty of the stimulus (e.g., by enhancing strength of evidence for the distractor stimulus or the variance in the signal). For instance, Herce Castañón et al. (2019) asked observers to judge the mean orientation across an array of Gabor patches. They manipulated stimulus energy by both increasing the contrast of these patches and increasing variance of the orientations across the array. Similarly, Koizumi et al. (2015) asked observers to pick which of two gratings that were superimposed on each other had the higher contrast. They manipulated stimulus energy by simultaneously increasing the contrast of both the target and the non-target gratings. These and similar types of energy manipulations are known to lead to confidence-accuracy dissociations, such that high stimulus energy leads to higher confidence despite accuracy being matched across energy levels (de Gardelle & Mamassian, 2015; Herce Castañón et al., 2019; Koizumi et al., 2015; Samaha et al., 2016; Spence et al., 2016; Zylberberg et al., 2014, 2016). For simplicity, in the rest of the paper we refer to stimulus features that increase stimulus discriminability as “contrast” and stimulus features that decrease discriminability as “variability” because these terms describe well most designs we examine in this study.

***Page 8

Paragraph 1
This paragraph could be finessed for clarity and readability
Does the term "positive evidence heuristic" meaningfully differ from confirmation bias? I did not find this term widely used in the literature or in the given citations.***

The positive evidence heuristic is distinct from confirmation bias since it is believed to be selective to perceptual confidence. Previous research has shown that while the perceptual choice is based on a balance of evidence between the chosen and unchosen options, confidence is based only on evidence for the chosen option (Maniscalco et al., 2016, 2021; Peters et al., 2017; Zylberberg et al., 2012). In contrast, confirmation bias refers to conditions where the evidence collected for the primary decision itself is based on prior beliefs about the correctness of the decision. The term “positive evidence” has been widely used in the confidence literature and we now clarify this term and cite more papers that use this term. We paste the relevant section below:

The most popular explanation is the positive evidence heuristic which assumes that confidence selectively neglects evidence that disconfirms the observer’s choice while the choice is based on a balance of evidence between all choice options (Maniscalco et al., 2016, 2021; Odegaard et al., 2018; Peters et al., 2017; Samaha et al., 2016; Samaha & Denison, 2022; Zylberberg et al., 2012).

***The description of noise blindness and how it works is not clear.***

We have now expanded on the noise-blindness explanation. We paste the relevant section below for reference:

Alternatively, these dissociations have been explained by assuming that humans infer decisions from suboptimal internal models which can result in over-confidence. Particularly, Herce Castañón et al. (2019) suggested that when observers integrate information across multiple sources (such as an array of Gabor patches), their internal computations are affected by cognitive noise arising from the process of evidence integration. However, observers are “blind” to this cognitive noise, resulting in an internal model that overestimates the quality of evidence and, hence, gives rise to over-confidence. Similarly, Zylberberg et al. (2014) have proposed that observers are insensitive to stimulus variance which also results in an internal model that fails to fully adjust confidence to account for increasing levels of uncertainty, resulting in overconfidence.

***Paragraph 2
It is not clear why energy manipulations would “naturally” lead to higher confidence***

By “naturally”, we meant in the absence of any additional mechanisms but have now removed this term to avoid confusion.

***The authors should be careful to distinguish signal detection theory (which is something that can characterize data) from actual mental representations. Signal detection is quite limited in terms of providing representational or mechanistic explanations.***

We thank the reviewer for this suggestion. We now clarify that the models we are referring to here are based on SDT assumptions about evidence distributions and criteria.

On the other hand, a simpler, SDT-based explanation for these effects has also been posited. According to this explanation, energy manipulations lead to changes in the distributions of evidence at the perceptual level, leading to higher confidence (Fetsch et al., 2014; Morales et al., 2015; Rahnev et al., 2011, 2013; Rahnev, Maniscalco, et al., 2012; Webb et al., 2023; Zylberberg et al., 2016).

***Greater input contrast to any neuron that is filtering for a pattern at any level of hierarchy
in a network will probably cause greater differentiation on the output. It’s not just limited to low level filtering. In a network like a CNN the increased contrast would likely propagate through the whole network.***

We agree with the reviewer that greater contrast would likely lead to greater discriminability regardless of the level of processing of the input. However, energy manipulations consist of contrast changes that happen simultaneously with changes in the variability of the stimulus. Further, these changes do not lead to changes in the discriminability of the input (as seen by the fact that d’ remains stable). Therefore, it is not a priori clear how energy manipulations should affect neural networks. We have now expanded substantially on how energy manipulations work (see our response to the first comment above regarding p.7) and believe that this clarifies the issue.

***Page 9

I am not at this point convinced that a signal detection model sheds any light at all on whether this effect is low level or high level (or a third alternative that it is some combination thereof). There needs to be much better support for the claim that a signal detection characterization corresponds solely to low-level processing. Why would this be the case? What is the proposed relationship to low-level mechanisms?***

After considering this point carefully, we agree with the reviewer that the argument for CNNs providing a method to distinguish between low- and high-level mechanisms is not very convincing, particularly since there is a lack of strong evidence linking the mechanisms of SDT models or CNNs to bottom-up vision in humans. Therefore, we have now removed all claims about SDT models and CNNs allowing us to capture low-level or bottom-up processes in humans.

***Are these two "explanations" perhaps not explanations at all? One is a well-known effect and the other is a computational theory-level mathematical characterization (at best). It's true that neither provides a specific mechanistic explanation, however.***

We agree with the reviewer that the signal-and-variance-increase hypothesis does not provide a mechanistic explanation for why changes in stimulus energy lead to changes in evidence distributions. However, the positive evidence (PE) bias apart from being associated with the behavior effect shown here, has been instantiated in independent mechanistic models of confidence (Maniscalco et al., 2016, 2021; Odegaard et al., 2018; Peters et al., 2017; Samaha et al., 2016; Samaha & Denison, 2022; Zylberberg et al., 2012). Therefore, the PE bias can be considered a mechanism by itself. We now add a paragraph in the Discussion to clarify that the signal-and-variance-increase model offers a characterization of the observed behavior in terms of internal evidence distributions but does not specify how the stimulus manipulations themselves lead to changes in evidence. We now emphasize this point in the Discussion and paste the relevant section below:

… Our current findings corroborate these findings and extend them by adding energy manipulations to the list of factors that can produce confidence-accuracy dissociations via the signal-and-variance-increase process. However, it must be noted that this process by itself does not provide a mechanistic explanation for why changes in stimulus energy should lead to changes in the properties of the evidence distributions. Further research is required to characterize the mechanisms responsible for the observed relationships between stimulus features and the perceptual evidence they generate.

***Paragraph 3: There are other possible reasons that a CNN would fail to mimic human behavior other than a lack of a built in positive evidence heuristic. For example, CNNs don't work like the human visual system.***

We agree with the reviewer that CNNs being unable to mimic humans can also be likely due to differences in how they process visual stimuli. However, in the Introduction, our aim was to state the specific implications of our findings for the positive evidence bias and noise-blindness mechanisms. Nevertheless, we discuss these considerations in more detail in the Discussion and point out that a failure to mimic human behavior can also result from differences in how networks process stimuli. We paste the relevant section below:

Future studies can test the proposed cognitive mechanisms underlying other kinds of confidence-accuracy dissociations against suitable alternative explanations involving only changes to perceptual evidence to gain insight into the true mechanisms of confidence. However, findings of CNNs failing to replicate human behavior cannot automatically be interpreted as evidence for the necessity of the cognitive mechanism being tested. Differences in behavior between humans and ANNs can also arise from fundamental differences in how their networks process the stimuli themselves (Wichmann & Geirhos, 2023).

***Page 10

This section could use some finessing for clarity and style/vocabulary.
'anticipate' is probably the wrong word here.***

We have removed this term from the section and edited it for clarity.

***“In addition, they reproduced another signature of confidence that is popularly regarded as evidence for the positive evidence bias (Maniscalco et al., 2016)” What signature is this?***

We have now clarified that the signature we are referring to is a dissociation between metacognitive sensitivity (meta-d’) and stimulus sensitivity (d’). We paste the relevant section below:

In addition, they reproduced a dissociation between metacognitive sensitivity and stimulus sensitivity, which is another signature of confidence that is popularly regarded as evidence for the positive evidence bias (Maniscalco et al., 2016).

***“Importantly, these observations highlight the common mechanisms underlying the behavior of humans and artificial systems, suggesting that these confidence accuracy dissociations may be driven by external features of the environment, without the need for specialized internal cognitive mechanisms.” It seems like it would be very difficult to make this claim using a CNN, again given that we know CNNs are not really anything like human vision. A more modest claim is made at the end of the paper, which is that 1) prior SDT research may have reached inappropriate conclusions and 2) a CNN can act as an existence proof that a network *may* not need high level processing (but that what humans do is another question).***

We agree with the reviewer and have now modified our conclusions in line with the reviewer’s suggestions. We paste the relevant section below:

These results demonstrate that CNNs exhibit human-like dissociations between confidence and accuracy, showing that it is indeed possible for networks to generate these behaviors in the absence of mechanisms such as the positive evidence heuristic and cognitive inference. Importantly, these observations shed light on the possibility of common mechanisms driven by external features of the environment underlying the behavior of biological and artificial neural networks.

***Page 11

Nowhere in this paper am I really getting a feel for how well the models really mimic human data for these tasks. I think this is important for evaluating claims about humanlike visual discrimination. For example, is the performance level of 70% an arbitrary decision, or does it correspond to something in the prior experiments?***

We made the decision to fix the performance level at 70% to avoid floor and ceiling effects on performance and confidence. Specifically, targeting an average accuracy of 70% would allow an error rate at which confidence will be informative. In our prior experiments, we have used staircasing to fix participants’ performances to 70% for the same reason (Shekhar & Rahnev, 2018). However, we did not try to directly match the networks’ accuracies to the accuracies observed in humans for the corresponding tasks since the task paradigms used in these studies included other factors such as cuing and manipulation of difficulty levels along with stimulus energy levels which makes direct comparison of accuracies difficult.

Nevertheless, we have checked the mean accuracy levels that were targeted in these prior experiments and found that Herce Castanon et al (2019) indeed obtained a mean accuracy of around 70% across the low- and high-stimulus energy conditions. On the other hand, Koizumi et al. (2015) obtained mean accuracy levels of 84% across the two energy conditions. However, in their experiment, each energy condition also included a manipulations of difficulty levels. Although they do not report the accuracies obtained for each individual difficulty level and energy condition, the mean accuracy for the difficult condition can be inferred to be below 84% since the mean across the easy and difficult conditions was 84%. We now justify our choice for fixing accuracy at 70% in the Results. We paste the relevant section below:

The stimulus parameters were chosen such that increasing energy levels resulted in the same average performance level of ~70% across the 25 instances of each network architecture. We targeted a mean accuracy of 70% in order to avoid floor and ceiling effects on performance while allowing an error rate at which confidence will be informative.

***Page 12

Figure 1 Caption
Clockwise and counterclockwise doesn't specify orientation (any orientation can be reached via clockwise or counterclockwise rotation--even from a 12:00 (vertical) reference point).***

We have clarified that the stimuli are oriented clockwise or counter-clockwise from the vertical. The edited section from the figure caption is pasted below:

In all three experiments, the task involved two-choice discrimination between stimulus configurations oriented clockwise and counterclockwise from the vertical.

***Paragraph 1: By ‘average’ to you mean the mean? Or another measure of central tendency?***

We have clarified that we are referring to the mean.

***Page 13

Figure 2
It's very hard to compare what the different models are doing when they are not plotted on the same scale.***

We have now plotted all the models on the same scale in Figure 2 to allow comparisons between them. We paste the modified figure below:

**Figure 2. Confidence-accuracy dissociations in CNNs.** For all experiments and networks (custom 4-layer CNN, VGG-19 and ResNet-50), accuracy was matched across energy conditions but confidence significantly increases with energy levels. The violin plots show the kernel density estimates of the data distribution. *p<0.05; **p<0.01; ***p<0.001; ****p<0.0001; n.s., not significant.

***Page 14

Strike 'highly significant' and just use ‘significant’. It's not a measure of the importance of the result.***

We have changed all instances of “highly significant” to “significant.”

***Again, I am looking for more detail about the match to the human data given the claims that the network is matching that data. Can you reproduce their figures/plot their data to make this comparison more clear?***

As mentioned earlier, the task paradigms used for the human studies were different from ours in ways that do not allow direct comparisons. For instance, the task in Koizumi et al (2015), was a 2x2 factorial design consisting of manipulations of positive evidence (evidence for the target grating) and difficulty (difference in contrast between the target and distractor gratings). Further, confidence was collected on a 4-point scale by Koizumi et al (2015) which would not allow for comparisons of confidence between the networks and humans. The task in Herce Castanon et al (2019) was also designed in a 2x2 factorial manner with stimulus contrast and tilt variability as factors and participants were presented with cues at the beginning of each trial which indicated which stimulus category was more likely to be presented. In our simulations, we simplified these designs to be able to directly study effect of stimulus energy manipulations. Therefore, it would be difficult to make any direct comparisons between these tasks. Our main objective was not to match human behavior but to test whether CNNs can reproduce the observed qualitative pattern of behavior where confidence increases with stimulus energy in spite of there being no changes in accuracy. We have now discussed these differences in task paradigms in the Methods and paste the relevant section below:

The task paradigms for Experiments 1 and 2 were adapted from Herce Castañón et al. (2019) and Koizumi et al. (2015). Both of these papers found confidence-accuracy dissociations in humans where confidence was found to increase with stimulus energy levels. However, we modified their original tasks with the purpose of isolating the effects of stimulus energy. Specifically, the task in Herce Castañón et al. (2019) was designed in a 2x2 factorial manner with stimulus contrast and tilt variability as factors and participants were presented with cues at the beginning of each trial that indicated which stimulus category was more likely to be presented. The task in Koizumi et al. (2015) was also a 2x2 factorial design consisting of manipulations of both positive evidence and task difficulty. In our simulations, we simplified these designs by excluding the cue manipulation in Experiment 1 and the difficulty manipulations in Experiment 2. We also replaced the 2x2 factorial design with a paradigm where we jointly varied stimulus contrast and variability across three levels. Due to these differences, it is difficult to make direct comparisons of accuracy between the networks and humans. Nevertheless, our networks achieved accuracies of 70% that that were comparable to humans in these tasks (humans showed an average of 70% in Experiment 1 and 84% in Experiment 2) and our results show that our task paradigms were indeed able to isolate energy manipulations and replicate their effects. To test the generality of our findings, we also included a novel task paradigm as Experiment 3 that has not been previously tested on humans but nevertheless uses the same kind of energy manipulations.

***“These findings cast doubt on the necessity of high-level explanations…”
I agree that doubt is cast on prior accounts, but I disagree with the general theme of the paper that that these results differentiate between high and low level processing in people. The output nodes in CNNs typically correspond to some sort of high-level object labeling, which a lot of researchers take to model high level vision at least, and maybe even language and cognition. True, there is no explicit built in confirmation bias mechanism in a CNN, but I still have reservations that a CNN is even a good approximation of the human visual system in the first place. In the human visual system, backprojections abound, for example. Vision is not feed-forward and low level processing is not truly independent of high level processing (or lateral interactions).***

We have removed all claims regarding these results allowing us to distinguish between low- and high-level hypotheses and modified the above sentence as follows:

These findings cast doubt on the necessity of the positive evidence and noise-blindness hypotheses for explaining confidence.

***Page 15

“While findings of confidence-accuracy dissociations in CNNs support the signal-and variance-increase hypothesis” I think this is probably too strong a claim if the idea is that it applies to people.***

We have removed the claim that these findings support the signal-and-variance-increase hypothesis and modified the section as follows:

The finding of confidence-accuracy dissociations in CNNs suggest that the positive evidence and noise-blindness hypotheses are unnecessary to explain these effects. However, to test whether the alternative signal-and-variance-increase process underlies this behavior, we examined how changes in stimulus energy affect the CNNs’ internal evidence distributions.

***“We accessed the CNNs’ internal representations by aggregating the activations generated in the networks’ output layer…” The output layer is not low level in a CNN, though. How is this an internal evidence representation having anything to do with low level processing? Furthermore, even if you were actually looking at internal representations (hidden layers), you can't really point to any node in a network with distributed representations like a CNN (with the exception of the inputs or outputs) and say that it corresponds to any specific representation, so I am skeptical that any specific representation in these networks could be probed at all. CNNs just don’t help us understand people.***

We agree with the reviewer that the distribution of evidence in the output layer does not necessarily correspond to low-level internal representations of the stimuli. Therefore, we have now removed all instances where we refer to the network’s output activations as “internal representations”. The modified sentence is pasted below:

We aggregated the activations generated in the networks’ output layer in response to images for each stimulus category separately for each energy condition.

***Page 16

Strike ‘highly significant’ and just use ‘significant’ for the reasons explained above.***

We have changed all instances of the term “highly significant” to “significant.”

***Report stats APA style with details of the test and statistic values.***

We have now reported the t-statistic and p-values associated with comparisons of mean confidence, separability of evidence and SD of evidence between the low and high stimulus energy conditions in Supplementary Table 1. We also clarify in the main text as well as the Supplementary that these are two-sided paired t-tests. We paste the table below for reference:

**Supplementary Table 1. Comparisons of mean confidence, separability and variance of evidence distributions between the low and high stimulus energy conditions for the three networks (4-layer CNN, VGG-19 and ResNet-50) across the four experiments.** Stimulus energy manipulations consists of simultaneous manipulations of contrast and variability in the same direction. Comparisons using two-sided, pairwise t-tests showed that confidence as well as separability and variance of the evidence distributions increased significantly from low to high stimulus energy condition for all networks and all experiments.

| **Network** | **Experiment** | **Confidence** | | **Separability of evidence** | | **Variability of evidence** | |
| --- | --- | --- | --- | --- | --- | --- | --- |
|  |  | **t(24)** | **p** | **t(24)** | **p** | **t(24)** | **p** |
| **4-layer CNN** | 1 | 6.25 | <0.0001 | 18.66 | <0.0001 | 20.36 | <0.0001 |
|  | 2 | 7.55 | <0.0001 | 18.86 | <0.0001 | 17.27 | <0.0001 |
|  | 3 | 14.99 | <0.0001 | 9.60 | <0.0001 | 11.05 | <0.0001 |
|  | 4 | 12.80 | <0.0001 | 28.57 | <0.0001 | 26.23 | <0.0001 |
| **VGG-19** | 1 | 11.59 | <0.0001 | 12.98 | <0.0001 | 11.98 | <0.0001 |
|  | 2 | 13.20 | <0.0001 | 11.16 | <0.0001 | 10.42 | <0.0001 |
|  | 3 | 6.59 | <0.0001 | 13.04 | <0.0001 | 13.49 | <0.0001 |
|  | 4 | 9.02 | <0.0001 | 7.41 | <0.0001 | 4.94 | <0.0001 |
| **ResNet-50** | 1 | 6.01 | <0.0001 | 21.21 | <0.0001 | 15.25 | <0.0001 |
|  | 2 | 12.91 | <0.0001 | 8.11 | <0.0001 | 7.86 | <0.0001 |
|  | 3 | 4.71 | <0.0001 | 7.25 | <0.0001 | 7.95 | <0.0001 |
|  | 4 | 9.43 | <0.0001 | 3.74 | 0.001 | 3.88 | 0.0007 |

***Page 17

I still feel that I need to better understand the human data given the number of comparisons in the paper. We have a not-great model of human vision that is supposed to be simulating human data, but we don't really know whether it does that well in the first place, from what is reported here.***

As mentioned earlier, the objective of this study was to test whether CNNs can reproduce the behavioral effect reported by Koizumi et al (2015) and Herce Castanon et al (2019). By manipulating stimulus energy in two distinct stimulus and task paradigms (explained in the introduction), they found that confidence increased with stimulus energy in spite of matched accuracy levels. The objective of this study is not to match or simulate human behavior but to test whether, in principle, such an effect can also be found in systems that do not explicitly contain the mechanisms hypothesized to be responsible for them. Therefore, for our purposes, it is more important to isolate the experimental manipulation that generated this effect rather than exactly match the network’s accuracies to human accuracies. Nevertheless, we have included a detailed explanation for why we have modified the task paradigms and mentioned that these changes do not allow direct comparisons between humans and networks. We pasted the relevant section under our response to the second comment for p.14 above.

***Here the scales are also not the same between plots.***

In this case, we believe that it does not make sense to match the scales on the plots because each network represents evidence on its own unique internal axis that is not comparable between networks. Matching the scales would mask differences of smaller magnitude (for instance in Experiment 3, for ResNet-50 the evidence ranges from -5 to +5 whereas for the 4-layer CNN, the evidence ranges from -20 to +20). We have now justified in the figure caption why the network activations have been plotted on different scales and paste the section below:

For all three experiments, the separability between the distributions of internal evidence for the two stimulus categories, as well as the variance of the evidence distributions, increased with energy levels. For each network, the figure shows the distance between the S_1 and S_2 evidence distributions and their standard deviations (SD) across the 25 model instances. We note that these networks appear to represent evidence on their own unique internal axis. Therefore, to optimally visualize differences in their evidence distributions, each network’s distributions have been plotted on their own unique scale.

***While one would probably expect that showing the network greater contrast would cause the separation reported here at the output of the CNN, it is not made explicit how this relates to human behavior or representation.***

We would like to clarify that we are not relating the changes in CNN outputs to models of human perception. We are only investigating how the network’s output distributions change due to stimulus energy manipulations and whether these changes are consistent with the signal-and-variance-increase hypothesis posited by SDT. We have now clarified this point in the manuscript and paste the relevant section below:

These findings show that the changes in the CNN’s internal evidence distribution are indeed consistent with the signal-and-variance increase hypothesis.

***Page 18

Use APA style. What is the exact statistical test, over what exact data?***

We have now clarified that the statistical tests conducted were pairwise t-tests and reported the full results of these tests in Supplementary Table 1 (pasted in the response above).

***“The concurrent increase in separation between the two stimulus categories and the
variability of evidence ensures that the network’s overall stimulus sensitivity remains
constant between the three energy conditions…” This sounds like the signal detection model is having an effect on the CNN, so the sentence should be changed for clarity. (The point is clarified in the next sentence.)***

We have rephrased these sentences to be independent of SDT terminology. The relevant section is pasted below:

The concurrent increase in separation between the evidence for the two stimulus categories and the variability of evidence ensures that the networks’ overall ability to discriminate between the two stimulus classes remains constant between the three energy conditions. Specifically, any improvement in the networks’ performance yielded by the increased separation between the evidence distributions for the two categories is counteracted by the evidence itself becoming more variable and confusable between the two classes. Nevertheless, confidence differences still emerge between conditions because the higher variance and separation between these distributions results in larger proportions of evidence being pushed towards extreme values that get assigned higher confidence. Together, these findings show that the confidence-accuracy dissociations produced by the CNNs can indeed be explained by the signal-and-variance-increase hypothesis.

***“These findings show that the signal-and-variance-increase mechanism indeed underlies the confidence-accuracy dissociations produced by the CNNs.” This is misleading because there is no "signal and variance increase mechanism" in a CNN. The SDT characterization of the output nodes changes, but that is not a mechanism in the network.***

We have now rephrased this sentence to refer to the “signal-and-variance-increase” as a hypothesis instead of a mechanism. The relevant section is pasted below:

Together, these findings show that the confidence-accuracy dissociations produced by the CNNs can indeed be explained by the signal-and-variance-increase hypothesis.

***Page 19

Earlier in this paper 'energy' was defined according to characteristics like contrast and variability, but now contrast and variability are being compared to energy. The manipulation needs to be clarified.***

We apologize for the confusion. We want to clarify that the “energy” of a stimulus always consists of two independent variables being manipulated simultaneously in the same direction. For example, the energy of a stimulus can be increased by jointly increasing both its contrast and noise levels. Energy cannot be manipulated by changing any single variable. However, in this section of the paper, we test how the networks respond to changes in each individual variable that constitutes the energy manipulation separately, while keeping the other variable fixed. The idea behind these analyses is to understand how each individual stimulus feature affects the characteristics of evidence, independent of the other. We have now clarified this point in the main text and paste the relevant section below:

While changes in the evidence distributions can explain the differences in confidence between energy conditions, it is still unclear why energy manipulations have this effect on the evidence distributions. Energy manipulations consist of two independent stimulus variables being manipulated simultaneously in the same direction such as its contrast and variability. To understand each variable’s individual effect on the networks’ internal evidence distributions, we manipulated each variable separately (while keeping the other fixed). We then compared these changes to the changes in evidence distributions observed during energy manipulations.

***Once again it seems strange to use the term 'internal evidence distributions' given that it's characterized over the output.***

We have removed all references to internal representations. However, we believe that the term “internal evidence distributions” makes sense because the activations generated by the network in the final layer can be thought of as the evidence it has generated for a given choice. This activation precedes the classification response and is therefore called an “internal” activation. We have now added this clarification to the Results and paste the section below:

We aggregated these activations across all instances of the network separately for all images from each energy condition. We then visualized the distributions of these activations separately for images from each stimulus category using kernel density plots.

***The reporting of the t-test should include what exact data was compared and use APA style with t statistics.

Page 20 - 21
Completely report statistical tests using APA style***

We have now completely reported the results of the t-tests for the independent manipulations of contrast and variability in Supplementary Table 2. We paste the table below for reference:

**Supplementary Table 2. Comparisons of separability and variance of evidence distributions when noise and variability are manipulated independently for the 4-layer CNN across the three experiments.** For contrast manipulations, the stimulus contrast was increased while stimulus variability was held constant. For variability manipulations, the stimulus noise or variability was increased while stimulus contrast was held constant. Comparisons using two-sided, pairwise t-tests showed that separability of evidence distributions increased significantly from the low- to high-contrast condition whereas the variance of evidence distribution decreased from the low- to high-contrast conditions for all experiments except Experiment 3. For Experiment 3, increasing contrast increased the variance of evidence distributions. On the other hand, separability of evidence distributions decreased significantly from the low to high variability condition and the variance of evidence distribution increased from the low to high variability conditions for all experiments.

| **Manipulation** | **Experiment** | **Separability of evidence** | | **Variability of evidence** | |
| --- | --- | --- | --- | --- | --- |
|  |  | **t(24)** | **p** | **t(24)** | **p** |
| **Contrast** | 1 | 15.33 | <0.0001 | -7.64 | <0.0001 |
|  | 2 | 16.91 | <0.0001 | -4.94 | <0.0001 |
|  | 3 | 21.71 | <0.0001 | 5.041 | <0.0001 |
| **Variability** | 1 | -7.74 | <0.0001 | 20.05 | <0.0001 |
|  | 2 | -15.07 | <0.0001 | 16.91 | <0.0001 |
|  | 3 | -27.56 | <0.0001 | 7.31 | <0.0001 |

***“Overall, these results demonstrate that manipulations of stimulus contrast and variability
have opposite effects on the separability and spread of internal activations…” I can believe this claim, but I am not sure how it relates to the question this paper aims to investigate. This does characterize CNN responses to changes in input characteristics, but it doesn't really speak to mechanism or level of representation in people.***

We want to clarify that we are not making any claims about representations in the human brain using these results. Due to the differences in visual processing between CNNs and humans, as pointed out by the reviewer, we cannot relate these results to human behavior or human visual representations. We are only trying to understand how changes in different stimulus features constituting the energy manipulations can affect the distributions of the network’s activations. We have now clarified this point in the main text and paste the section below:

Overall, these results demonstrate that manipulations of stimulus contrast and variability have opposite effects on the separability and spread of internal activations in CNNs, such that contrast manipulations have a larger effect on separability and variability manipulations have a larger effect on spread. Thus, combining both manipulations in a single “energy” manipulation in these networks leads to both increased separability and spread of the internal evidence distributions.

***Page 22

“So far, our results have shown that CNNs, in spite of lacking the positive evidence
mechanism, can produce human-like confidence-accuracy dissociations…” given the lack of detail about the human data this is probably too strong a claim about the network producing ‘human-like’ results.***

We would like to clarify that by “human-like” we mean that the CNNs produce the same pattern of energy-induced confidence-accuracy dissociations CNNs as seen in humans. Nevertheless, we have rephrased this sentence as follows:

So far, our results have shown that CNNs, in spite of lacking the positive evidence mechanism, can produce the kind of stimulus-energy induced confidence-accuracy dissociations that have typically been attributed to the positive evidence heuristic in humans.

***Consider reordering the sentences about type 1 and type 2. I wasn't sure which kind of type-1 and type-2 the authors were referring to at first.***

We have re-written this section to clarify the definitions of stimulus and metacognitive sensitivities. The relevant section is pasted below:

Another feature of confidence that has been attributed to the positive evidence mechanism is the observation that under certain conditions, an observer’s stimulus sensitivity (ability to discriminate between stimulus classes) and metacognitive sensitivity (ability to discriminate between correct and incorrect responses using confidence) are found to dissociate from each other under certain experimental designs (Maniscalco et al., 2016). Using SDT, one can quantify stimulus sensitivity as the amount of information available for the primary stimulus judgement (d’), and metacognitive sensitivity as the amount of information underlying confidence judgements (meta-d’).

***Page 23

“Overall, our findings demonstrate that for at least for some stimulus manipulations, low-level mechanisms are sufficient to explain effects that have typically been taken as evidence for a positive evidence mechanism.” This is an interesting result with respect to the prior claims using SDT models as evidence for the necessity of a certain kind of high-level processing. This is relevant to researchers using SDT models. I am still unconvinced that the alternative account should be called ‘low-level mechanisms’ given what we know about human visual processing, and the fact that CNNs don’t reveal much about how they represent information.***

We have rephrased this sentence to remove claims about low-level mechanisms as follows:

Nevertheless, our findings demonstrate that for at least for some stimulus manipulations and networks, the positive evidence mechanism is unnecessary to explain effects that have typically been considered as evidence for this mechanism.

***Page 24

“Our previous findings establish that low-level changes in perceptual representations can
explain human behavior that has typically been attributed to high-level cognitive
mechanisms. Here, we sought to further test whether the “robust averaging” mechanism
can also be realized through low-level mechanisms” The previous findings established that changes in inputs to CNNs produce patterns in a signal detection framework that have previously been attributed in other signal detection modeling to high level cognitive mechanisms. While this casts doubt on prior SDT work, this a reminder that there is not a real connection to the human visual system’s representations yet. There are no actual mechanisms specified by this SDT framework.***

We have now rewritten this section to remove references to low- and high-levels mechanisms. The revised section is pasted below:

Our findings thus far establish that simple changes in the separation and variance of stimulus evidence distributions can explain human behavior that has typically been attributed to cognitive mechanisms such as the positive evidence bias and noise-blindness. Here, we sought to further test whether the “robust averaging” mechanism can also be realized through such changes in evidence distributions.

***Page 25

Strike ‘highly significant’ and replace with ‘significant’ for reasons previously stated.***

We have removed all instances of the term “highly significant”.

***“Nevertheless, these results while establishing the generalizability of the mechanisms underlying confidence-accuracy dissociations in CNNs also reveal how stimulus-specific interactions can constrain the similarities between humans and CNNs.” This sentence should be reworded for clarity. I’m not really sure what the claim is.***

We have now rephrased this section for clarity and paste the section below:

Nevertheless, these results establish the generalizability of the signal-and-variance-increase process underlying confidence-accuracy dissociations in CNNs. Further, they suggest that while for some stimuli (such as the Gabor patches tested here), the signal-and-variance-increase hypothesis can possibly explain the behavior of both humans and CNNs, for certain other stimuli (such as colors), the behavior of CNNs and humans diverge likely due to human visual decision-making relying on additional, stimulus-specific mechanisms that CNNs lack.

***Page 27

“Our findings support the alternative, signal-and-variance increase hypothesis which posits that such dissociations naturally emerge from low-level changes in perceptual representations.” This is too strong a claim. The authors’ findings support the idea of a relationship between the energy characteristics on the input layer of a CNN and the distribution of activity on the output layer, and that prior SDT research might have come to incorrect conclusions about certain types of high level accounts of this effect.***

We agree with the reviewer and have now revised this claim as follows:

…our findings support the idea that changes in the internal evidence distributions are sufficient to account for such dissociations.

***“…could be driven by common, stimulus-driven processes and demonstrate the usefulness of CNNs in distinguishing between low- and high-level explanations of behavior.” This statement is not really supported by this work, again because CNNs are very different than the human visual system in terms of their architecture particularly recurrency and backprojection. CNNs by virture of their simplistic architecture can’t speak to high level and low level interactions. The link to what is actually known about human visual representation is missing.***

We have removed the claim that CNNs allow us to distinguish between low- and high-level explanations of behavior and paste the revised version below:

These findings suggest the possibility of common, stimulus-based processes driving the behavior of both artificial and biological systems and demonstrate the usefulness of CNNs in testing the necessity of specific computational explanations of human behavior.

***Page 28

This is a great passage and in my opinion is really what this paper is about: evidence against prior SDT conclusions. The prior PE literature perhaps had some flaws that led to poor conclusions about human visual discrimination and representation, in part because data characterized by SDT models might have many different kinds of underlying mechanisms, and SDT does not generally specify a mechanism.***

We thank the reviewer for the positive comment.

***Page 30

“..observers’ failure to scale their decision and confidence criteria in response to low-level
perceptual changes” Be careful here. Some low level changes are not cognitively penetrable, but others are. It's not clear that anything can be said about decision criteria on low-level stimuli subject to noise. Again, there is constant interaction between top-down and bottom-up processing in the real visual system.***

***Unless you're analyzing the internal layers of the CNN, it's still quite strange to refer to 'internal representations'***

We agree with the reviewer and have now removed the reference to the low-level changes. We have also changed the term “internal representations” to “sensory evidence”. The modified paragraph is pasted below:

… it must be noted that overconfidence and base-rate neglect both arise from the observers’ failure to scale their decision and confidence criteria in response to changing levels of uncertainty in stimulus evidence. In that sense, this effect might reflect a blindness to changes in the properties of evidence at the decision stage. Nevertheless, this mechanism is distinct from the one proposed by Herce Castañón et al. (2019) because in their model the blindness is towards noise arising from the observers’ own internal cognitive processes, rather than towards noise arising from stimulus-driven changes in sensory evidence.

***The TMS data is interesting, but it’s coming quite late in the paper, and the same issues with CNNs apply. The link between the TMS, the SDT, and the CNN is tenuous.***

We refer to the TMS findings to show that, like stimulus energy manipulations, TMS can also induce confidence-accuracy dissociations where confidence is influenced independent of changes in accuracy. More importantly, these findings were explained via SDT modelling which showed that these dissociations were associated with changes in the separation and variance of stimulus evidence distributions. These findings support the notion that confidence-accuracy dissociations can be produced solely via changes in the properties of evidence in the absence of any cognitive explanations such as PE and noise-blindness. We have now clarified the link between the TMS findings, SDT and our findings. We have also removed references to low-level changes and internal representations.

… The notion that confidence can be influenced solely by changes in sensory evidence is not new. Brain stimulation applied to lower (Rahnev et al., 2013; Rahnev, Maniscalco, et al., 2012) and mid-level visual areas (Fetsch et al., 2014) has been found to affect confidence, independent of changes in accuracy. These effects were captured well by SDT models where stimulation increased the variance of internal evidence. Other task manipulations involving attention (Morales et al., 2015; Rahnev et al., 2011) and evidence volatility (Zylberberg et al., 2016) also produced similar dissociations in confidence that were accounted by SDT modelling via changes in the trial-by-trial variance of sensory evidence. Our current findings corroborate these findings and extend them by adding energy manipulations to the list of factors that can produce confidence-accuracy dissociations via the signal-and-variance-increase process.

***“Several recent studies have argued that deep neural networks can provide meaningful
insights into the goals and constraints that have shaped human perception..” Bowers et al. (2023) argued that they cannot. Bowers et al. (2023). Deep problems with neural network models of human vision. Behavioral and Brain Sciences, 46, e385***

We thank the reviewer for pointing us to this reference. We have now added a paragraph discussing Bowers et al.’s arguments against using CNNs as models of human vision and as asked by another reviewer, describe how our current study could inform this question. The paragraph from the Discussion is pasted below:

Our study can also help inform the debate on whether CNNs can serve as useful models of human vision. Bowers et al. (2023) argue against the notion that CNNs can be applied as models of human perception since these models have not been adequately tested on their ability to account for findings in cognitive science research. Although CNNs perform visual classification tasks with high levels of accuracy, it is not yet understood whether these networks process stimulus features in ways similar to humans. Bowers et al. argue that to establish such similarity in processing, it is important to test CNN’s behavior on manipulations of independent stimulus features and compare their behavior to humans on psychological tasks. In this study, we follow a similar logic as we manipulate stimulus features such as contrast and variance and find that CNNs indeed respond similarly to humans under the specific stimuli tested here. Further, these manipulations change the properties of the CNNs’ evidence distributions in a way that is consistent with the predictions of an empirically validated model of perception, suggesting that, in the case of the stimuli tested here, a common mechanism can possibly explain the behavior of both humans and ANNs.

***“Indeed, our findings support this argument as they carry implications about the external
constraints that may have shaped visual processing.” This is too much of an overclaim. CNN's cannot capture the majority of human behavior in vision. Again, see Bowers et al. (2023)***

We would like to clarify that we do not intend to claim that CNNs are able to capture human behavior in general. However, as Bowers et al. (2023) have argued, it is important to manipulate stimulus features independently and compare how humans and CNNs process such simple features and the behaviors they generate. We find that both humans and CNN models not only produce the same qualitative behavior in response to energy manipulations but there is also evidence (via SDT modelling in humans) that their internal evidence distributions may respond to these manipulations in similar ways. These findings suggest that these two systems may be processing simple stimulus features such as contrast and variance in similar ways. One possible explanation for this similarity between two the kinds of networks is that these common mechanisms are being driven by the stimulus feature itself, rather than some mechanism internal to the network. We have now clarified this point in the main text and paste the relevant section below:

Several recent studies have argued that deep neural networks can provide meaningful insights into the goals and constraints that have shaped human perception (Blauch et al., 2021; Cao & Yamins, 2024; Dobs et al., 2022; Doerig et al., 2023; Gomez-Villa et al., 2019; Kell et al., 2018; Kell & McDermott, 2019; Richards et al., 2019; Wichmann & Geirhos, 2023). Our findings carry implications about the external constraints that may have shaped visual processing. For instance, we find that both humans and CNN models produce the same behavior in response to energy manipulations and, further, the same signal-and-variance-increase process posited to work in humans underlies the behavior of CNNs. One possible explanation for the similarity in feature processing between the two networks is that the processing is being driven by the stimulus feature itself, rather than by some mechanism inherent to the network.

***“Another critical advantage of CNNs is that they can allow us isolate bottom-up visual processes from the top-down mechanisms that serve cognition, since visual processing in standard CNNs occurs in the absence of specialized top-down influences.” Again, this is an overclaim. In this paper the authors have demonstrated that CNNs can show that there is a network that exists that can produce the behavior that prior researchers claimed was only possible with specific top-down influences.***

We have now removed the claims about CNNs allowing us to isolate bottom-up and top-down processes.

***“In addition, they can inform us about the possible stimulus and task representations that
underlie such bottom-up stimulus processing (Green et al., 2024).” This sentence seems disconnected from the rest of the paper.***

We have also removed this sentence since it refers to the claim about using CNNs to understand bottom-up processing.

***Page 33

“These findings cast doubt on the necessity of invoking high-level, cognitive explanations for this phenomenon – particularly the popular assumption that confidence is derived from a positive-evidence heuristic.” This is a much more measured and accurate claim that is still scientifically important, especially to researchers using SDT.

“Our results highlight the necessity of disentangling low- and high-level explanations of behavior and establish CNNs as promising models for generating and testing hypotheses about the mechanisms underlying human behavior.” This work does not really establish the usefulness of CNNs for understanding human vision. It does establish the usefulness of CNNs for disproving prior claims about human vision in SDT modeling.***

We have modified this claim as follows:

Our results highlight the necessity of disentangling low- and high-level explanations of behavior and establish CNNs as promising models for testing the necessity of cognitive explanations of human behavior.

***Page 34
“The task was to decide whether the average tilt across the 8 patches was clockwise (CW) or counterclockwise (CCW) from the horizontal (Figure 1A).” What is the range of possible orientations? In principle could it wrap all the way around? Probably not but what’s the limit?***

The tilt of the eight patches was sampled from a Gaussian distribution such that the orientations. In principle, it is possible for the tilts to wrap around 360 degrees. We have now mentioned this in the main text:

The possible range of orientations was $\left[ 0, 2\pi\right]$.

***Page 35

Same as page 34 with range of possible orientations.***

For the other two experiments, there was no range of possible orientations as they were fixed at 45 degrees. We now mention this explicitly in the Methods. (As we mentioned in our response to the comment above, for Experiment 1, we have now mentioned that the possible range of orientations was $\left[ 0, 2\pi\right]$.)

**Reviewer #3 *The positive evidence bias for confidence (PE) refers to the intriguing observation that confidence in the accuracy of a decision appears to depend more on evidence supporting the chosen option than on evidence supporting non-chosen alternatives. Despite extensive research, the cause of PE bias remains unknown. The manuscript entitled "Human-like Dissociations Between Confidence and Accuracy in Convolutional Neural Networks" examines the emergence of PE bias in CNNs under manipulations of stimulus energy. The study questions the need for "high-level" cognitive mechanisms to explain PE bias, and presents evidence for the signal-and-variance-increase hypothesis through extensive experimentation with different CNN architectures. I found the paper to be well written and interesting, and I recommend publication pending the authors addressing the concerns outlined below.***

We thank the reviewer for the helpful comments and suggestions.

***Comments:

1.
An important contribution of the paper is that, unlike the study by Webb et al. in 2023, confidence is not explicitly trained, but rather read-out from a network trained to perform different classification tasks. It would be beneficial to clarify early in the results section how confidence is computed from the CNN output, since this is pivotal to the paper and sets it apart from previous studies. That is, explain that a final layer with one node (activated by sigmoid) is added to the networks, and confidence is computed as a rectification of this activation.***

We have now clarified in the Introduction as well as Results that confidence was computed as the output of the final layer of the network transformed by a sigmoid activation function. We paste the relevant sections below:

… In each experiment, we manipulated the energy of the stimulus by simultaneously varying two independent stimulus features in the same direction: the contrast of the stimulus and its variability (in Experiments 1 and 3) or the contrast of the stimuli associated with the correct and incorrect choices (in Experiment 2). As standard in the literature (Guo et al., 2017), confidence was computed as the output of the final layer transformed by a sigmoid activation function.

***2.
The validity of the study rests on the assumption that the confidence readout from the CNN accurately models human confidence judgments. However, some aspects of the data suggest that this is not a valid assumption. In many of the reported experiments, confidence levels approach ceiling values, suggesting that they may have little discriminative power with respect to decision accuracy. For example, in Experiment 1 (Figure 2), the mean confidence exceeds 0.95 across all energy levels, suggesting that confidence has minimal ability to discriminate between correct and incorrect decisions.

The findings would be more meaningful if the authors could identify operational settings for these networks where confidence resolution and calibration more closely mirror those observed in human experiments for similar tasks.***

We agree with the reviewer that it is important to check the calibration and resolution of confidence in CNNs and ensure that they are meaningful. Following the reviewer’s suggestion, we have now included two additional analyses to the Supplementary. Firstly, we test whether confidence generated by our networks can discriminate between correct and incorrect choices. Our findings show that confidence in these networks is significantly higher for correct choice compared to incorrect choices for all networks and across all energy levels, confirming that these networks indeed generate meaningful confidence that tracks their accuracy. Second, we use a calibration method called “temperature-scaling” which uses a scaling parameter that penalizes overconfidence and matches confidence to accuracy. Indeed, this method matched confidence to accuracy levels for all networks but still robustly produced the confidence-accuracy dissociations. We paste the results from these analyses below:

**Supplementary Figure 1. Assessing resolution and calibration of confidence**. (A) We tested whether the CNN’s confidence could reliably discriminate between correct and incorrect choices for the different energy conditions. We found that for all stimulus energy levels and all three networks (4-layer CNN, VGG-19 and ResNet-50) confidence was higher for correct choices compared to incorrect choices, confirming that the networks’ confidence reliably tracked their accuracies. (B) CNNs tend to be overconfident and generate confidence values greater than their accuracies. Therefore, we tried to calibrate the CNNs’ confidence using a post-processing method called “temperature-scaling” (Guo et al., 2017) which uses a parameter $(T)$ that can scale the network’s confidence to match its accuracy. The network’s output in logits ($z$) is scaled before the sigmoid transformation such that $z_{scaled}=\frac{z}{T}$. Confidence is then computed as $\frac{1}{1+e^{-\frac{z}{T}}}$ and therefore, when $T>1,$the network’s confidence gets scaled down. We found that temperature scaling indeed reduced overconfidence in all networks (4-layer CNN: confidence decreased from 0.968 to 0.737; VGG-10: confidence decreased from 0.964 to 0.783, ResNet-50: confidence decreased from 0.982 to 0.748). However, for all networks we still robustly observed the confidence-accuracy dissociations.

***3.
I found the distinction drawn between "high-level" and "low-level" explanations of PE bias not particularly helpful. The authors suggest that CNN outputs are considered "low-level," while additional processes are deemed "high-level." They argue for a low-level explanation of PE bias based on their findings, but I remain uncertain about this conclusion.***

We agree with the reviewer’s point that the claims regarding CNNs allowing us isolate low- level processes from cognitive or high-level processes is too broad. We also agree that since CNNs cannot yet be considered good models of the brain, our results cannot be extended to processes occurring in the brain. Following the reviewer’s suggestion, we have now removed all claims about CNN’s allowing us to distinguish between low- and high-level processes. We now limit our conclusions to the specific mechanisms (the positive evidence bias, noise blindness and signal-and-variance-increase) we test in this study. Additionally, we also emphasize the point that our results cannot falsify the positive evidence bias and noise-blindness mechanisms in humans. Rather, we only show that these mechanisms, in spite of their popularity, are not necessary to explain these observed dissociations. We have now made edits throughout the paper to reflect these changes. Below, we paste some of the edited portions from the Abstract and Introduction as examples:

Abstract

These findings cast doubt on the necessity of the positive evidence heuristic to explain human confidence and establish CNNs as promising models for testing cognitive theories of human behavior.

Results

… Here, we use convolutional neural networks (CNNs) to distinguish between these competing explanations of the confidence-accuracy dissociations induced by stimulus energy manipulations. Standard CNNs give confidence using the same signal as the decision, and thus do not use the positive evidence heuristic (where decision-incongruent evidence is ignored during confidence computations). They also give confidence without building internal models of the task. Therefore, if the positive evidence heuristic (Koizumi et al., 2015; Maniscalco et al., 2016; Peters et al., 2017; Samaha et al., 2016) or inference from suboptimal internal models (Herce Castañón et al., 2019; Zylberberg et al., 2014) are indeed necessary for these dissociations, these networks should fail to mimic human behavior. On the other hand, if confidence-accuracy dissociations arise from a signal-and-variance increase based on inherent stimulus or task characteristics (Fetsch et al., 2014; Morales et al., 2015; Rahnev et al., 2011, 2013; Rahnev, Maniscalco, et al., 2012; Zylberberg et al., 2016), we can expect neural network models to reproduce human behavior. An additional advantage of CNNs is that, unlike humans, we can directly probe the network’s internal representations and understand the mechanisms underlying their behavior.

…These results demonstrate that CNNs exhibit human-like dissociations between confidence and accuracy, showing that it is indeed possible for networks to generate these behaviors in the absence of mechanisms such as the positive evidence heuristic and cognitive inference. Importantly, these observations shed light on the possibility of common mechanisms driven by external features of the environment underlying the behavior of biological and artificial neural networks.

***The authors define confidence as a transformation of activation in the CNN's final layer. This transformation might be viewed as "high-level" because it involves an additional operation on the network's output rather than being directly performed by the neural network itself.
In the studt, confidence is assumed to be the distance between “a” and the decision threshold. Because this transformation is unaffected by stimulus noise, it is, in my view, identical to models of confidence that assume "noise insensitivity," a concept the authors regard as "high-level." Had the authors employed a transformation sensitive to noise, the observed PE bias might not have emerged. If so, then the critical factor for observing the PE bias is how confidence is read-out from the activation “a”.

My specific suggestions here are:
(i) Be more explicit about what is meant by “low level” and “high level”, such that it is clear why the signal-and-variance-increase hypothesis is considered “low level” and the noise blindness hypothesis “high level”. I think the paper would be better without framing it in terms of “low” vs “high” levels, but of course that is up to the authors.***

As suggested by the reviewer, we have completely removed claims about the signal-and-variance-increase hypothesis being low level and the PE and noise-blindness hypotheses being high-level. We refer to these hypotheses by their names and do not make claims about the level of processing involved.

***(ii) Explore if a mapping from activation “a” to confidence that does take noise into account eliminates the PE bias. If so, the PE bias still depends on the readout mechanism, as postulated by “high level” theories.***

We agree with the reviewer that a high-level theory postulating the existence of a mechanism that adjusts confidence criteria according to noise levels can potentially eliminate the observed confidence-accuracy dissociations. However, this finding would still suggest a lack of an additional inference process that that informs the observer about the level of uncertainty associated with the stimulus and allow appropriate adjustment of confidence criteria.

Nevertheless, as suggested by the reviewer, we have now removed all general claims regarding these dissociations being independent of high-level mechanisms. Instead, we now limit our claims to the specific mechanisms of noise-blindness and positive evidence bias. Specifically, while our findings do not rule out the necessity of all high-level theories, they show that these specific theories are not necessary to explain energy-induced confidence-accuracy dissociations.

***4.
One issue with showing the “violin” plots with individual data points is that highly statistically significant differences may not be visually apparent (e.g., the confidence comparison for the VGG-19 network in Figure 2). To improve clarity, authors might consider keeping the "violin" plots to illustrate the distribution of the data, but show means with error bars instead of individual data points.***

We thank the reviewer for this suggestion. We have now modified all of our violin plots by removing the individual data points and replacing them with point plots showing the mean and SEM of the distributions. We paste all the revised figures below:

**Figure 2. Confidence-accuracy dissociations in CNNs.** For all experiments and networks (custom 4-layer CNN, VGG-19 and ResNet-50), accuracy was matched across energy conditions but confidence significantly increases with energy levels. The violin plots show the kernel density estimates of the data distribution. *p<0.05; **p<0.01; ***p<0.001; ****p<0.0001; n.s., not significant.

**Figure 3. Energy manipulations increase both the separability and variance of the networks’ internal activations.** For all three experiments, the separability between the distributions of internal evidence for the two stimulus categories, as well as the variance of the evidence distributions, increased with energy levels For each network, the figure shows the distance between the $S_{1}$ and $S_{2}$evidence distributions and their standard deviations (SD) across the 25 model instances. The kernel density plots show the distribution of activations aggregated across all 25 network instances. *p<0.05; **p<0.01; ***p<0.001; ****p<0.0001; n.s., not significant.

**Figure 4. Changes in the separation and spread of internal evidence distributions induced by energy, contrast, and variability manipulations.** The plots show A) the average distance between the mean activations for $S_{1}$ and $S_{2}$ stimuli (left) and B) the average standard deviation (SD) of activations (right) in the final layer of the shallow CNNs for Experiments 1-3 in response to changes in stimulus energy, contrast and variability. Note that the energy results in both panels are equivalent to the 4-layer CNN results from Figure 3. For all experiments, increasing energy and contrast levels increases the separation between the two stimulus categories, while increasing variability decreases the separability between the two stimulus categories. On the other hand, increasing stimulus energy and variability increases the spread of evidence distributions, while increasing contrast decreases the spread of evidence for all experiments except experiment 3 (where increasing contrast increases the spread of evidence). These results suggest contrast and noise changes selectively drive changes in separation and variance of evidence distributions respectively. The violin plots show the kernel density estimates of the data distribution. *p<0.05; **p<0.01; ***p<0.001; ****p<0.0001; n.s., not significant.

**Figure 6. Confidence-accuracy dissociations in a color discrimination task.** A) The stimulus consisted of an array of eight colored circles. The task was to determine whether the mean color across the eight patches was more blue or red. In this example, the mean color is more blue than red. Energy manipulations involved joint changes to the intensity of color (the amount of “blueness” or “redness” of the patches as well the variance in color across the array. (B) The separability between the stimulus categories as well as the variance of the evidence distributions increased with energy levels for all three networks. The panels on the top-left for each network show the average distance between the $S_{1}$ and $S_{2}$evidence distributions across the 25 model instances. The panels on the bottom-left show the average standard deviation (SD) across the two distributions across all model instances. The panels on the right show the distribution of activations aggregated across all 25 network instances. *p<0.05; 250 **p<0.01; ***p<0.001, ****p<0.0001; n.s., not significant.
