# Supplementary material for: Human-like dissociations between confidence and accuracy in convolutional neural networks
Source: PLoS Comput Biol. 2024 Nov 14;20(11):e1012578. doi: 10.1371/journal.pcbi.1012578 (PMC11594416; doi:10.1371/journal.pcbi.1012578)
Supplement: Attachment — Submitted filename: Rebuttal.docx [file pcbi.1012578.s006.docx]

Dear Dr. Doumas,

Thank you once again for giving us the opportunity to revise and improve our manuscript. We also thank Reviewer 2 for their helpful follow-up comments. Following the reviewer’s suggestion, we now explicitly refer to the network’s output as “activations” or “evidence” and have removed all mentions of the phrase “internal evidence”. We provide a point-by-point response to the reviewers’ comments below.

Sincerely,

Medha Shekhar and Dobromir Rahnev

**Reviewer #1**

***The revised version of this manuscript addresses all my previous concerns, therefore I recommend to accept.***

We thank the reviewer for the recommendation.

**Reviewer #2**

***I appreciate the author's responses and substantial revisions, which address many of the issues in the original version of the manuscript. The result is a stronger and clearer paper. The discussion section in particular is very strong, and the methods are clear. There are a few remaining minor issues.***

We thank the reviewer for the positive and helpful comments. We include a point-by-point response below.  ***Although the authors have clarified what they mean by "internal evidence" and "internal representations" (and I do see what they are trying to get at, which is that one can characterize what the network has encoded overall by examining responses on the output layer to various stimuli), I still believe the term 'internal' is potentially confusing in reference to the output layer.***

***For example, Page 7: "An additional advantage of CNNs is that, unlike humans, we can directly probe the network’s internal representations and understand the mechanisms underlying their behavior." sounds like probing the intermediate/hidden layers and making sense of them, which is practically impossible given the way such networks work, and is not what happened in this work.***

We agree with the reviewer that using the term “internal evidence” to refer to the network’s output can be potentially confusing. To avoid such confusion, we have now replaced all mentions of the term “internal evidence” with “evidence” or “activations” and explicitly mention that these evidence distributions refer to the activations in the networks’ output layer. Below, we paste some of the edited portions from the Abstract and Introduction as examples:

Abstract

…Further, we traced back the reason for the confidence-accuracy dissociations in all CNNs to the same signal-and-variance increase that has been proposed for humans: higher stimulus energy increased the separation and variance of evidence distributions in the CNNs’ output layer leading to higher confidence even for matched accuracy.

Introduction

…An additional advantage of CNNs is that, unlike humans, we can directly probe the network’s activations and understand the mechanisms underlying their behavior.

***Page 9: "As [is] standard in the literature"***

We thank the reviewer for catching this error. We have now fixed it. ***Page 12: Do the authors mean "from human studies" instead of "from human behavior"?***

We indeed meant to refer to previous studies on human behavior. We have now modified the sentence as follows.

…These results mimic findings from human behavioral studies where increasing the energy of the stimulus leads to increases in confidence, despite accuracies being matched across conditions

***Page 21: It would be nice to have some more insight than "interactions between the stimuli and the networks" when the effect isn't obtained. I don't insist, but if the authors have any insight as to why the effect does not always obtain, it might help to include it.***

Our previous statement explaining why we fail to observe the meta-d’/d’ dissociations for all stimuli and networks was indeed vague. However, at this point, we can only speculate that the meta-d’/d’ dissociations may depend partially on a different set of processes that are more sensitive to the specifics of the network and the stimuli. Further research is required to characterize these processes and examine why these dissociations, unlike confidence-accuracy dissociations, fail to generalize across network architectures and stimuli. Nevertheless, we have now explicitly discussed this point and paste the relevant section below:

… It is possible that unlike the confidence-accuracy dissociations examined above, meta-d’/d’ dissociations may partially rely on a different set of processes that are more sensitive to the specifics of the stimuli and network architectures. However, it is not yet clear what these processes may be and why meta-d’/d’ dissociations are less robust than confidence-accuracy dissociations. Further work is required to address these questions.

***Page 24: I don't understand the statement about "increases in confidence between levels"--perhaps this is a typo?***

We now clarify that the ANOVA revealed significant differences in mean confidence between the three stimulus energy levels, rather than an increase. The subsequent t-tests show that confidence increased for higher energy levels. We paste the relevant section below for reference:

…However, the ANOVA revealed significant differences in mean confidence between the three stimulus energy levels for all three networks

***Page 26: "Underling" should probably be "Underlying"***

We thank the reviewer for catching this typo. We have fixed now fixed it. ***Page 31: "Further, these manipulations change the properties of the CNNs’ evidence distributions in a way that is consistent with the predictions of an empirically validated model of perception, suggesting that, in the case of the stimuli tested here, a common mechanism can possibly explain the behavior of both humans and ANNs."

The only problem here is that we already know that CNNs fail to predict human behavior in many cases. So the phrase "common mechanism" makes perhaps too strong a commitment to some factor or mechanism internal to the network as opposed to something about the stimulus, a possibility the authors have already pointed out.***

We agree with the reviewer that the term “common mechanism” may be confusing since it can also refer to a mechanism internal to the networks. Indeed, our intention was to emphasize that observing similarities in the behavior of humans and ANNs does not automatically imply the existence of a common internal mechanism underlying their behavior. Rather, given the known differences in visual processing between CNNs and humans, we argue that a more likely explanation is the existence of a common *external* factor (such as the statistical properties of the stimuli) that drives their internal processing. We have now modified this statement to further clarify what we mean:

…Further, these manipulations change the properties of the CNNs’ evidence distributions in a way that is consistent with the predictions of an empirically validated model of perception. We suspect that, in the case of the stimuli tested here, a common external factor, such as the statistical properties of the stimuli themselves, drive the behavior of both humans and ANNs. If so, this suggest that in tasks where performance is strongly driven by the structure of the stimuli themselves, CNNs may provide a useful model of human perceptual decision making.

***Page 32: "However, findings of CNNs failing to replicate human behavior cannot automatically be interpreted as evidence for the necessity of the cognitive mechanism being tested. Differences in behavior between humans and ANNs can also arise from fundamental differences in how their networks process the stimuli
themselves (Wichmann & Geirhos, 2023)." These sentences were very hard to follow.***

We have now expanded this paragraph to clarify what we mean. Specifically, we argue that while probing for the necessity of a cognitive mechanism, a failure of CNNs to produce the associated behavior cannot be automatically interpreted as evidence in favor of that cognitive mechanism in humans. Due to there being fundamental differences in how humans and CNNs process even low-level visual features, it becomes difficult to interpret such differences and attribute them to a single cognitive factor. Evidence from other sources that directly test the necessity of this mechanism in humans (using causal interference or cognitive modelling) becomes necessary to corroborate such findings. We have pasted the relevant section below:

However, in the case that CNNs fail to mimic human behavior, one cannot automatically infer that the mechanism being tested is necessary to produce that behavior. Such divergence in the behavior of humans and CNNs can alternatively be explained by fundamental differences in how the two systems process visual stimuli themselves (Wichmann & Geirhos, 2023).

***Page 36: "The possible range of orientations was [0, 2]." switches to radians when everything else was in degrees.***

We have edited all references to the orientations to consistently refer to them in degrees.

**Reviewer #3**

***The authors have addressed all my concerns. Congratulations to the authors for a very interesting paper.***

We thank the reviewer for the positive comment.
